# Fatal laryngeal burn from ingestion of a hot fish cake: Case report and literature review

**DOI:** 10.1016/j.ijscr.2020.04.056

**Published:** 2020-05-14

**Authors:** Michael M.H. Chu, Antonia Tse, Ileana Anderco, Arun Cardozo

**Affiliations:** Lancashire Teaching Hospitals NHS Foundation Trust, Sharoe Green Lane, Fulwood, Preston PR2 9HT, United Kingdom

**Keywords:** Larygeal burn, Fatal, Thermal epiglottitis, Hot food, Case report

## Abstract

**Introduction:**

Laryngeal burn from hot food ingestion is a rare but potentially life-threatening presentation. It is essential that clinicians have a high index of suspicion of potential airway obstruction in such cases. To our knowledge, this is the only case of fatal laryngeal oedema caused by hot solid food ingestion reported in the literature.

**Presentation of case:**

A 51 year old male presented to the Emergency Department complaining of a burn to the throat following ingestion of a piping hot fish cake. On initial assessment he reported only mild pain and increased saliva production. There was no evidence of stridor, dysphagia, dyspnoea or aspiration and hence the patient was discharged and advised to return if he experienced any worsening of his symptoms. 2 h later the patient collapsed at home and died due to airway obstruction from delayed laryngeal oedema.

**Discussion:**

27 cases of laryngeal burn from ingestion of hot food or liquid were identified in the literature. Only one fatality following ingestion of hot liquid has been reported. This is the first documented fatal laryngeal burn due to ingestion of hot solid food. At present, there are no guidelines for the assessment and management of laryngeal contact burns.

**Conclusion:**

Patients may be relatively asymptomatic immediately after thermal injury to the larynx. Endoscopic examination of the larynx is required to identify laryngeal oedema. Any sign of thermal injury to the laryngeal mucosa warrants admission for observation and definitive management of the airway should be considered.

## Highlights

• Laryngeal burns from hot food ingestion are rare but potentially life-threatening.
• There is often a delay between thermal insult and progression of laryngeal oedema.
• Laryngeal oedema may occur in the absence of oropharyngeal signs of thermal injury.
• Clinicians should have a low threshold for referral to ENT for flexible laryngoscopy.
• Definitive airway management with intubation or tracheostomy may be required.

## 1 Introduction

Thermal burns to the oral cavity and oropharynx from hot food are very common and normally inconsequential. However, thermal injury to the larynx can cause potentially fatal laryngeal or supraglottic oedema. Oedema can progress many hours after the initial injury. Emergency physicians and junior grade Ear, Nose and Throat (ENT) doctors are likely to be the clinicians seeing this presentation in the first instance. Thus, it is essential for frontline clinicians to recognise the seriousness of this presentation and be familiar with the initial assessment and management. This case has been reported in line with the SCARE criteria [1].

## 2 Presentation of case

A 51-year old Caucasian male self-presented to the Emergency Department of his local District General Hospital complaining of a burn to the throat following ingestion of a piping hot fish cake. On arrival he was triaged, and deemed suitable for review in the Urgent Care Centre to be seen by a General Practitioner or Advanced Nurse Practitioner. Of note, the patient had suffered an ischaemic stroke 7 years prior, and spent 18 months in stroke rehabilitation. Despite rehabilitation, the patient was left with a baseline moderate speech deficit and occasionally aspirated upon swallowing. He was assessed by an Advanced Nurse Practitioner and at that time his speech was deemed to be at baseline fluency. The patient noticed mild dysphonia which he reported this to his partner, but not to the assessing clinician. He complained of an increase in saliva production. He denied any odynophagia, dysphagia, shortness of breath or history of aspiration. On examination, his observations were all within normal limits. There was no stridor and auscultation of his chest was unremarkable. His case was discussed with the ENT Senior House Officer (SHO) on-call based at the nearby Teaching Hospital. Given the patient was stable and relatively asymptomatic, he was discharged and advised to return if there was any worsening of his symptoms.

2 h later the patient complained of increasing throat pain to his partner. He felt unwell and went to lie down. 15 min later he called out for his partner then subsequently collapsed at home.

His partner initiated cardiopulmonary resuscitation (CPR) following advice from the emergency services advisor over the phone. On arrival, the ambulance first responder performed an emergency tracheostomy at the scene. CPR was on-going on arrival to the Emergency Department. The electrocardiogram (ECG) demonstrated ineffective cardiac activity and after 25 min CPR was abandoned and the patient was confirmed deceased.

**Fig. 1:**
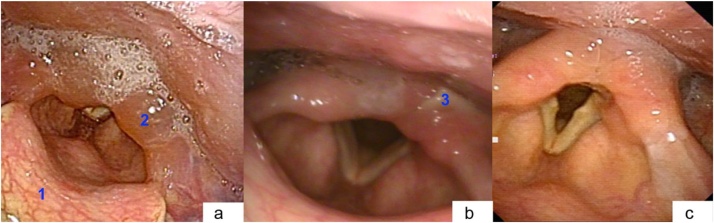
Images adapted from (2) with permission. (Patient presented in this case report did not have endoscopic evaluation.) Views from flexible laryngoscopy in 81 year old male following ingestion of microwaved meat stew. Image a: Initial examination following thermal contact burn. 1 = normal epiglottis anteriorly. 2 = mucosal oedema of arytenoids with pooling of saliva posteriorly. Image b: Four days post-injury. 3 = White plaques of healing mucosa in arytenoid region. Image c: Healed mucosa at one week follow up.

Post-mortem examination revealed a swollen epiglottis and significant swelling of the left aryepiglottic fold. There was no oedema of the true or false vocal cords and the glottic airway was patent. The tracheostomy was inserted appropriately below the cricoid cartilage. A lack of mast cells or eosinophils in the tissues suggested the swelling was not secondary to anaphylaxis. It was concluded that cause of death was asphyxia secondary to delayed swelling of the upper airway due to thermal injury (Fig. 1).

## 3 Discussion

Minor burns to the oral cavity from ingestion of hot food or beverages are common. As a reflex, the hot food or drink is usually expelled immediately. These burns are often mild and managed conservatively. However, in rare cases thermal burns can be fatal if they extend deeper to affect the hypopharynx and larynx. To date, there have been 27 reported cases of laryngeal burn secondary to hot food or beverage ingestion dating back to 1977 (2–26). Burns following hot beverage consumption were more frequently reported (*n* = 16) than burns associated with hot food (*n* = 11). To our knowledge, this is the first fatal laryngeal burn caused by hot solid food ingestion reported in the literature. There has been one reported case of a fatal burn sustained following ingestion of a hot beverage in a psychiatric patient [15].

Nearly half of all reported cases (*n* = 13) involved children and almost all of these involved hot liquid ingestion [[4], [5], [6], [7], [8], [9], [10], [11], [12], [13],^19^,^20^]. Particularly with young children, clinicians should be aware that laryngeal burns can be sustainted following facial scald burns and subsequent steam inhalation, without actual ingestion of the hot liquid [27,28].

In adults, mental ill-health may also be a risk factor for laryngeal burns following hot food or drink ingestion [3,15,17,18]. Indeed, the one fatal larygeal burn in the literature occured when a schizophrenic patient rapidly drank a cup of hot coffee. 6 h later he developed respiratory distress. His combative behaviour prevented appropriate evaluation and he suffered a respiratory arrest [15].

In the case presented here, the injury was sustained following ingestion of a hot fish cake which came fresh out of the deep fryer. It is unclear whether his previous stroke had resulted in an oropharyngeal sensory deficit which allowed the patient to swallow the piping hot food item. Interestingly, the use of dentures may reduce oral heat perception, allowing contact laryngeal burns to occur [2].

Microwave heated food and drink was implicated in 7 of the cases reported in the literature [2,4,5,10,14,16,24]. The dielectric heating mechanism of microwaves results in heterogenous heating of food. This creates internal “hot spots” within the food item, allowing the food to bypass the oropharynx and subject the epiglottis or larynx to an intense thermal insult [24].

Currently, there are no guidelines for the assessment and management of laryngeal contact burns. However, there is a wealth of literature on inhalation injury in the context of burns management. The International Society for Burn Injuries (ISBI) have produced a guidance document which includes recommendations for inhalation injury [29]. Assessing and protecting the airway in a thermally injured patient is paramount. Clinicians are advised to have a high clinical suspicion for the development of airway obstruction and a low threshold for intubation in burns patients. Our case report would suggest that contact laryngeal burns from hot food should also be treated with a similar degree of suspicion. Clinical signs such as stridor, dysphonia, drooling and bilstering of the oropharyngeal mucosa are suggestive of impending airway obstruction. Our patient’s previous stroke had left him with a baseline speech deficit. This may have made it difficult for the assessing clinician to recognise new dysphonia suggestive of laryngeal oedema.

Crucially, the development of airway oedema following thermal injury may be delayed in onset. The timing and severity of airway oedema is difficult to predict accurately. Airway obstruction occurs when oedema develops in the epiglottis and supraglottic airway [30]. Indeed, some papers call this phenomenon ‘thermal epiglottitis’ [6,8,12,17,18,28]. Maximal oedema usually occurs between 8 and 36 h after the inital insult, and lasts for up to 4 days [30,31]. It is often seen following aggressive fluid resuscitation, more so in the context of patients who have also sustained concurrent cutaneous burns [30].

Fibreoptic evaluation with flexible nasendoscope is essential to definitively diagnose supraglottic oedema. Normal endoscopic appearance of the larynx can be reassuring in patients where the history and clinical signs are suggestive of a thermal injury to the larynx. However, due to the potential delayed onset of laryngeal oedema these patients should be observed and repeat fibreoptic evaluation is warranted, particularly if there is clinical deterioration.

In confirmed upper airway burns, patients should be nursed in a semi-upright position to improve venous and lympahtic drainage, thus reducing airway oedema [29]. Endotracheal intubation is indicated if airway compromise occurs. In the infective epiglottitis literature, prophylactic intubation in children is recommended if there is any sign of airway compromise [32]. Accordingly, 12 of the 13 reported paediatric cases of thermal epiglottitis were indeed intubated (See Table 1). Tracheostomy is only indicated if the patient cannot be intubated due to swelling, or when prolonged mechanical ventilation is anticipated [33].

**Table 1:** Cases of thermal burns to the larynx caused by food or beverages reported in the the literature.

Lead Author (ref)	Year	Adult / Paeds	Steroids	Antibiotics	Intubation	Tracheostomy	Food / Drink	Confounding Factors	Adverse Outcome
Jung [3]	1977	adult	–	+	–	+	coffee	mental health	
Sando [4]	1984	paeds	–	+	–	–	milk	microwave	
Garland [5]	1986	paeds	+	+	+	–	tea	microwave	
Kulick [6]	1988	paeds	+	+	+	–	beverage		
Brahams [7]	1989	paeds	+	–	+	–	tea		brain damage
Laufkoetter [8]	1989	paeds	+	+	+	–	vegetables		
Dye [9]	1990	paeds	–	+	+	–	tea		
Dye [9]	1990	paeds	–	–	+	–	water		
Goldberg [10]	1990	adult	+	+	–	–	potato	microwave	
Mazrooa [11]	1990	paeds	+	–	+	–	tea		
Harjacek [12]	1992	paeds	–	+	+	–	tea		
Williams [13]	1993	paeds	+	+	+	–	tea		
Ford [14]	1994	adult	+	+	–	–	treacle tart	microwave	
Mellen [15]	1995	adult	–	–	–	–	coffee	mental health	death
Offer [16]	1995	adult	+	–	+	–	jacket potato	microwave	
Kornak [17]	1996	adult	–	+	–	–	tomato	mental health	
Ma [18]	1996	adult	+	+	–	–	stewed tomato	mental health	
Watts [19]	1996	paeds	–	–	+	–	tea		
Lai [20]	2000	paeds	+	+	+	–	water		
Goto [21]	2002	adult	+	+	–	–	milk	intoxication	
Alpay [22]	2008	adult	+	+	–	–	water		
Shenoy [23]	2009	adult	+	+	–	–	food		
Silberman [24]	2013	adult	+	–	+	–	lasagne	microwave	
Iyama [25]	2016	adult	–	–	+	–	water		
Hyo [2]	2017	adult	+	+	–	+	bun	microwave, dentures	
Hyo [2]	2017	adult	+	+	–	–	meat stew	dentures	
Inaguma [26]	2019	paeds	+	+	+	–	tofu		
Chu	2020	adult	–	–	–	–	fish cake	previous stroke	death

Interestingly, studies from the burns literature suggest that corticosteroids and antibiotics should not be given in the inital treatment of thermal inhalation injuries [29,34]. It is not clear whether this applies to the management of airway oedema secondary to laryngeal contact burns. However, in most of the reported cases the patients were given both intravenous (IV) steroid and empirical antibiotics (see Table 1). The likely rationale is that these patients were treated in the same manner as one would manage acute infectious epiglottitis. There is no high quality evidence showing that corticosteroids reduces the need for intubation, duration of intubation or duration of hospital stay in the acute epiglottitis literature. However, one retrospective cohort study showed that IV steroid use reduced length of hospital stay in acute epiglottitis managed on the intensive care unit (ICU) [35].

## 4 Conclusion

In summary, we report the first case of a patient who died from asphyxia secondary to delayed laryngeal oedema as a result of thermal injury to the larynx from rapid ingestion of a hot food item. This is an extremely uncommon mechanism of sustaining a laryngeal burn and highlights the perils of thermal injury to the upper aerodigestive tract, regardless of how innocuous the insult may seem. Endoscopic examination of the larynx is essential to rule out laryngeal oedema. Any sign of thermal injury to the laryngeal mucosa warrants admission for observation and definitive management of the airway should be considered.

## Declaration of Competing Interest

Nothing to declare.

## Funding

Nothing to declare.

## Ethical approval

This study is exempt from ethical approval in our institution. This is not an original research study.

## Consent

Written informed consent was not obtained from the patient. The head of our medical team has taken responsibility that exhaustive attempts have been made to contact the family and that the paper has been sufficiently anonymised not to cause harm to the patient or their family. A copy of a signed document stating this is available for review by the Editorin-Chief of this journal on request.

## Author contribution

Michael Chu: conceptualization, writing - original draft, visualization.

Antonia Tse: writing - review and editing.

Ileana Anderco: writing - review and editing.

Arun Cardozo: conceptualization, supervision, project administration, writing - review and editing.

## Registration of research studies

1. Name of the registry: n/a.
2. Unique identifying number or registration ID: n/a.
3. Hyperlink to your specific registration (must be publicly accessible and will be checked): n/a.

## Guarantor

Michael Chu – lead author.

Arun Cardozo – supervisor.
